# Smarcd1 Inhibits the Malignant Phenotypes of Human Glioblastoma Cells via Crosstalk with Notch1

**DOI:** 10.1007/s12035-020-02190-z

**Published:** 2020-11-14

**Authors:** Yihao Zhu, Handong Wang, Maoxing Fei, Ting Tang, Wenhao Niu, Li Zhang

**Affiliations:** 1 Department of Neurosurgery, Jinling Hospital, Medical School of Nanjing University, 305 East Zhongshan Road, Nanjing, 210002 Jiangsu People’s Republic of China; 2 Department of Neurosurgery, The Affiliated Brain Hospital of Nanjing Medical University, Nanjing, 210029 Jiangsu People’s Republic of China

**Keywords:** Smarcd1, Glioblastoma, Proliferation, Migration, Chemoresistance, Notch1 signaling pathway

## Abstract

Smarcd1 is a component of an evolutionary conserved chromatin remodeling complex—SWI/SNF, which is involved in transcription factor recruitment, DNA replication, recombination, and repair. Suppression of the SWI/SNF complex required for cellular differentiation and gene regulation may be inducible for cell proliferation and tumorigenicity. However, the inhibitory role of Smarcd1 in human glioblastoma cells has not been well illustrated. Both U87 and U251 human glioblastoma cell lines were employed in the present study. The lentivirus-mediated gene knockdown and overexpression approach was conducted to determine the function of Smarcd1. The protein levels were tested by western blot, and the relative mRNA contents were detected by quantitative real-time PCR. Cell viability was tested by CCK-8 and colony-forming assay. Transwell assays were utilized to evaluate the motility and invasive ability. Flow cytometry was employed to analyze cell cycle and apoptosis. SPSS software was used for statistical analysis. Low expression of Smarcd1 was observed in glioblastoma cell lines and in patients with high-grade glioma. Importantly, the depletion of Smarcd1 promoted cell proliferation, invasion, and chemoresistance, whereas enhanced expression of Smarcd1 inhibited tumor-malignant phenotypes. Mechanistic research demonstrated that overexpression of Smarcd1 decreased the expression of Notch1, while knockdown of Notch1 increased the expression of Smarcd1 through Hes1 suppression. Hence, the crosstalk between Smarcd1 and Notch1, which formed a feedback loop, was crucial in regulation of glioblastoma malignant phenotypes. Furthermore, targeting Smarcd1 could be a potential strategy for human glioblastoma treatment.

## Introduction

Glioblastoma is the lethal primary brain tumor in the central nervous system (CNS). Despite aggressive treatments, namely accurate surgery accompanied by chemotherapy and radiotherapy, the prognosis remains dismal with a median survival of less than 20 months and patients with inevitable recurrence usually survive less than 12 months [1]. The 2016 World Health Organization classification of tumors in the CNS highlighted new entities of molecular features in defining the glioma grades [2]. Therefore, comprehensive studies at the cellular and molecular levels are urgently needed to find better therapeutic, diagnostic and prognostic targets of glioblastoma.

Smarcd1 is the non-catalytic subunit, but is indispensable in all the three final-form complexes (canonical BAF, polybromo-associated BAF, non-canonical BAF) of the SWI/SNF family [3, 4]. As previously reported, Smarcd1 harbored remarkable function in interacting with transcriptional factors, such as Tbx1 [5] and P53 [6]; and recruiting nuclear proteins, such as glucocorticoid receptor and AP1 [7]. In mice hepatic cells, Smarcd1 had a critical role in activating chromatin structure of the ROR response elements on the proximal Bmal1 and G6Pase promoters to facilitate transcription [8], thus regulating circadian clock and energy metabolism. Recent studies of Smarcd1 focused on modulating H3K27me3 redistribution on the chromatin to regulate pluripotency-associated factor KLF4 in embryonic stem cells [9]. Smarcd1 has been reported downregulated in several human malignant tumors [10, 11]. Hong and his colleagues demonstrated that the expression of Smarcd1 sensitized lung cancer cells to chemotherapy drug-induced apoptosis via the inhibition of miR-7 [10]. Nevertheless, Smarcd1 is also involved in cellular senescence and resistance to DNA damage [12]. However, whether or not the expression of Smarcd1 is dysregulated in glioblastoma is still unknown and the potential mechanism of Smarcd1 in regulating the malignant phenotypes of glioblastoma is urgent to be discovered.

Notch signaling regulates lineage differentiation, cell cycle progression, and self-renewal of stem cells [13], which is postulated to act as an oncogene in multiple cancer lines [14, 15], including certain types of brain cancer. Notch1 is deemed to be activated in primary glioblastoma, whereas low-grade astrocytomas seem to show an inactive Notch signaling [16]. Ahmad and his colleagues have found that knockdown of the core subunit of SWI/SNF complex, BRG1, significantly reduced the transcriptional activation of targeted genes [17], whereas Smarcd1 and BRG1 are evolutionary conserved and structurally integrated subunits in the SWI/SNF family. Taken together, these may lend to the hypothesis that Smarcd1 interacts with Notch1 signaling to regulate the malignant phenotypes.

In the present study, we investigated the tumor suppressor role of Smarcd1 in human glioblastoma in vitro and in vivo. The findings here demonstrated that overexpression of Smarcd1 reduced cell proliferation, invasion and chemoresistance of glioblastoma. The crosstalk between Smarcd1 and Notch1 contributed to the malignancy of glioblastoma and break up of this feedback loop may be a potential therapeutic target for glioblastoma treatment.

## Methods and Materials

### Glioma Tissues and Cell Lines

A total of 37 glioma samples and 11 specimens of normal brain tissues were collected from patients without anti-cancer treatments before surgery in Jinling Hospital. The non-tumor tissues were obtained from the non-functional brain parenchyma when resecting deep benign tumors or lesions. This study strictly abided by the Declaration of Helsinki and approved by the Ethics Committee of Jinling Hospital, and the involved patients signed informed consent forms before surgery.

U87 and U251 human glioblastoma cell lines were obtained from the Cell Bank of Type Culture Collection of the Chinese Academy of Sciences (Shanghai, China), which were cultured in Dulbecco’s modified Eagle’s medium (DMEM; Gibco, Waltham, MA, USA) supplemented with 10% fetal bovine serum (Thermo Fisher Scientific, Waltham, MA, USA) and 1% penicillin/streptomycin (HyClone, GE Healthcare Life Sciences, Logan, UT, USA). Human astrocytes (HA) were purchased from the Institute of Basic Medical Sciences (Beijing, China), and the growth medium was from ScienCell Research Laboratories (San Diego, CA, USA). The cells were incubated in a humidified atmosphere 5% CO_2_ at 37 °C.

### Reagents and Antibodies

DAPT (*N*-[*N*-(3,5-difluorophenacetyl)-L-alanyl]-S-phenylglycine t-butylester), TMZ (temozolomide), and Hes1 siRNA were purchased from Sigma-Aldrich (St. Louis, MO, USA). Lipofectamine 3000 reagent was bought from Thermo Fisher Scientific. The primary antibodies for western blot and immunofluorescence against cyclin D1, CDK4, Catenin, Vimentin, ZO-1, E-Cadherin, N-Cadherin, Bcl-xl, Bax, Caspase3, Cleaved Caspase3, BRG1, Notch1, and β-actin were purchased from Cell Signaling Technology (Danvers, MA, USA). The primary antibodies against Hes1 and Hey1 were purchased from Abcam (Cambridge, MA, USA). Smarcd1 primary antibodies were purchased from BD Biosciences (San Jose, CA, USA). The relative secondary antibodies for western blot and immunofluorescence were purchased from Jackson Laboratories (West Grove, PA, USA).

### siRNA Transfection

The relative siRNAs of Smarcd1 and Notch1 were synthesized in Transheep Technology (Shanghai, China). Si-Smarcd1-1: AUGAGGAAACGGCUAGAUATT; Si-Smarcd1-2: AGACGUGAAUGUACGGUGUTT; Si-Notch1: CGGGACAUCACGGAUCAUATT. siRNAs were diluted in DEPC (diethyl pyrocarbonate) water (Beyotime, Shanghai, China) into 20 μM. About 2 × 10^5^ cells were seeded into the 6-well plate for 24 h. Lipofectamine 3000 and siRNA were blended into mixture and then added into the DMEM (with 10% fetal bovine serum). After transfection, cells were incubated for 48 h and then harvested for further studies.

### Lentivirus Package and Infection

The knockdown and overexpression lentivirus were constructed in Hanbio Biotechnology (Shanghai, China), and the transfection protocol was followed the manufacturer’s instructions. Smarcd1 shRNA and non-targeted control sequence were cloned into the GFP-contained Lentivirus vector. The Smarcd1 sequence (NM_003076) was gained from NCBI to be subcloned into a plasmid lentiviral vector to overexpress Smarcd1, while an empty vector was utilized as a negative control. Cells were transfected with lentivirus for 48 h, and then, the efficiency of Smarcd1 knockdown and overexpression was analyzed.

### Cell Viability Assay

Cell viability was done with the Cell Counting Kit-8 (CCK-8; Dojindo, Kumamoto, Japan) following the manufacturer’s instruction. Equal amount of about 1 × 10^3^ cells was seeded in the 96-well plates and cultivated for 24 h and then treated with or without interventions for 3 days. After incubating within 10% CCK-8 dilutions for 2 h, the plates were analyzed with a Bio-Rad microplate reader at the value of 450-nm wavelength (OD450). All the tests were performed at least 3 times.

### Migration and Invasion Assay

To test the motility of glioblastoma cells treated with diverse interventions, the transwell analysis was operated. A total of 2 × 10^5^ cells in 200 μL DMEM were infused into the upper chamber of 6.5-mm transwells with 8.0-μm pore polycarbonate membrane inserts (Corning Incorporated, Corning, NY, USA). Additive matrigel matrix (Corning Incorporated) was laid over the upper chambers especially in the cell invasion assay. After incubating at 37 °C for 16 h, we cleaned up the cells in the interior of the chamber that did not penetrate the membrane. Then, the chambers were fixed with 4% paraformaldehyde for 15 min and then stained with 0.1% crystal violet for 10 min. The cells were observed in six microscopic fields (× 200) under an inverted microscope by two investigators blind to the grouping (Carl Zeiss Meditec AG, Jena, Germany).

### Western Blot

The elaborate procedure of western blot was described previously [18]. The BCA method was taken to detect protein concentrations. Equal amounts of protein were added to 10–12% SDS-PAGE gel and then transferred to polyvinylidene difluoride (PVDF) membranes (EMD Millipore, Billerica, MA, USA). The membranes were blocked with 5% skim milk-TBST for 90 min and then incubated in corresponding primary antibodies overnight at 4 °C. After washing in TBST, the membranes were incubated with HRP-conjugated secondary antibodies for 2 h at room temperature. Finally, the protein bands were exposed to a chemiluminescence imaging system (Tanon, Shanghai, China) with enhanced chemiluminescence detection reagents (EMD Millipore).

### Quantitative Real-Time PCR

Total RNA was extracted from glioma samples or cultured cells using Trizol reagent (Invitrogen, Carlsbad, CA, USA) following detailed protocol and then reverse transcribed cDNA was amplified. Relative forward and reverse primer sequences were listed as follows: 5′-GGAGACCGATGGCTTTCAGG-3′ and 5′-AGGGTCCTGGAGCTTATGTGTC-3′ (Smarcd1); 5′-GCAGTCAGGCGTGTTGTTCT-3′ and 5′-GGCACTTTCTGTGAGG-AGGAC-3′ (Notch1); 5′-ATTCTGGAAATGACAGTGAAGCAC-3′ and 5′-CACCTC-GGTATTAACGCCCTC-3′ (Hes1); 5′-TCGGCTCCTTCCACTTACTG-3′ and 5′-TTC-CCCTCCCTCATTCTACA-3′ (Hey1); 5′-TGCGAACCAAAGCGACCA T-3′ and 5′-GCCTTAGCATTGAGGGCTGTCT-3′ (BRG1); and 5′-CACCCAGCACAATGAAGAT-CAAGAT-3′ and 5′-CCAGTTTTTAAATCCTGAGTCAAGC-3′ (β-actin).

### Co-immunoprecipitation Assay

Cultured cells in a density of 70% were harvested in IP lysis buffer (Guge Bio, Wuhan, China) and centrifuged for total input protein in 4 °C. Anti-mouse IgG and A/G beads (Santa Cruz Biotechnology) were incubated in the protein lysate for 1 h and then centrifuged for supernatants. The Smarcd1 antibody was added for reaction in a rotator at 4 °C overnight with subsequent additional A/G beads for 2 h. After that, the immunoprecipitation sediments were harvested and then eluted with IP buffer 3 times. The input total protein and final immunoprecipitation products were analyzed by western blot.

### Immunofluorescence Assay

Different groups of U87 and U251 cells were fixed with 4% paraformaldehyde for 20 min while the glioma tissue sections were paraffin embedded. The slides were then treated with 0.5% Triton X-100 (Biofroxx Bio, Germany) for 10 min. The primary antibodies (anti-Smarcd1, anti-Notch1, or anti-Hes1) were incubated at 4 °C overnight following the CY3-conjugated or Alexa Fluor-488 secondary antibody for 1 h at room temperature. Nuclei were stained with DAPI (Sigma-Aldrich) for 10 min. All the images were taken using a ZEISS immunofluorescence microscope (× 400) by two investigators blind to the grouping.

### Flow Cytometry

Glioblastoma cells were harvested and then suspended by 500 μL binding buffer containing 5 μL of Annexin V-FITC (KGA108, KeyGEN, China) and 5 μL of propidium iodide (PI, BD Biosciences). The apoptotic cells were measured using a FACS Calibur flow cytometer (BD Biosciences).

### Immunohistochemical Staining

The xenograft tumor sections were incubated with anti-Ki67 antibody (Cell Signaling Technology) overnight at 4 °C. After 1× PBS washing for 2 times, the above sections were incubated with HRP-conjugated anti-rabbit IgG for 1 h. The targeted protein was stained with diaminobenzidine, and the nucleus was counterstained with hematoxylin. All the brain sections were observed in 10 cortical microscopic fields (× 400) and then counted.

### Tumor Xenografts Study

All experimental procedures and animal care were reviewed and approved by the Animal Ethics Committee of Jinling Hospital. Four-week-old male BALB/c nude mice were used to establish subcutaneous xenotransplanted GBM model. Xenograft tumor volume and weight were measured and recorded. The detailed procedure was described previously [19].

### Statistical Analysis

SPSS 23.0 (IBM, Armonk, NY, USA) was employed to perform statistical analyses. All data were expressed as mean ± SEM. One-way and two-way ANOVA were utilized in variance analysis among multiple groups, and Student’s *t* test was employed in comparison between 2 groups. *P* < 0.05 was regarded as significant difference.

## Results

### Smarcd1 Was Downregulated in Human Glioblastoma Tissues and Cell Lines

**Fig. 1:**
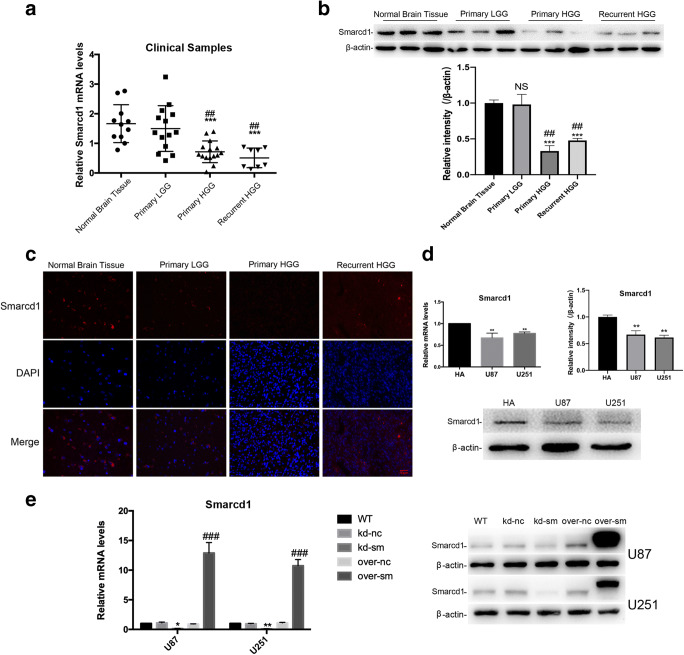
Smarcd1 was downregulated in human glioma tissues and glioblastoma cell lines. **a** 11 samples of normal brains and 37 samples of glioma tissues (LGG: 14 samples, primary HGG: 15 samples, recurrent HGG: 8 samples) were collected and then succumbed to qRT-PCR analysis. The expression of Smarcd1 on primary and recurrent HGG samples was significantly reduced than in LGG and normal tissues. No expression difference was detected between primary and recurrent HGG samples. **b**, **c** 3 samples of each groups above were randomly collected and the western blot (**b**) and immunofluorescence (**c**) results revealed the protein level of Smarcd1 was decreased compared with normal brain tissues. **b** The protein bands density of Smarcd1 and β-actin was measured by ImageJ software and then underwent statistical analysis, which showed that Smarcd1 in primary and recurrent HGG was significantly decreased than normal brain and primary LGG. The relative protein levels of control cells were adjusted to the value of 1. ***p < 0.001 versus normal brain tissue, ##*p* < 0.01 versus LGG. **d** The expression of Smarcd1 in glioblastoma cell lines (U87 and U251) was declined compared with HA cells, which were measured by PCR and repeated western blot densitometric quantification by ImageJ. **p < 0.01 versus HA cell. **e** Lentivirus-mediated Smarcd1 gene knockdown and overexpression were performed in U87 and U251 cells. The mRNA and protein levels of Smarcd1 were reduced after gene knockdown while boosted in the overexpression group as compared to relative control group. *p < 0.05, **p < 0.01 versus kd-nc group; ###*p* < 0.001 versus over-nc group. All data were represented as the means ± SEM of three independent experiments

To explore the role of Smarcd1 in human glioblastoma, we first determined its differential expression compared with normal brain tissues and astrocytes. Namely, 14 samples of low-grade glioma (LGG, WHO I and II) tissues, 15 samples of primary high-grade glioma (HGG, WHO III and IV) tissues, and 8 samples of secondary high-grade glioma tissues were taken to analyze the relative expression of Smarcd1. As shown in Fig. 1a, the mRNA levels of Smarcd1 varied in each group while tissues of LGG showed no significant difference compared with normal brain tissues. Notably, the expression of Smarcd1 in the primary and secondary HGG groups were significantly decreased than LGG and normal brain groups. However, there was no difference between primary and secondary HGG (Fig. 1a), which indicated that Smarcd1 had no influence on tumor recurrence according to our data. What’s more, we randomly took 3 samples of each group to detect Smarcd1 protein expression by western blot and immunofluorescence. As demonstrated previously, the protein level quantified by ImageJ software (Fig. 1b) and fluorescence intensity (Fig. 1c) in the primary and secondary HGG groups were much less than normal brain tissue and LGG.

Similarly, we employed qRT-PCR and western blot to analyze the relative expression of Smarcd1 between glioblastoma cell lines (U87 and U251) and human astrocyte (HA). The mRNA expression declined by 32% and 22% respectively in U87 and U251 cells compared with HA cells; western blot showed the same decreased protein expression in glioblastoma cells as well (Fig. 1d). Taken together, these results illustrated that the expression of Smarcd1 was downregulated in glioma tissues and cell lines and potentially exerted the tumor suppressor role.

To further investigate the bio-function of Smarcd1 in glioblastoma cells, lentivirus-mediated Smarcd1 gene knockdown and overexpression were employed in this study. The transfection efficiency was measured by qRT-PCR and western blot, and as shown in Fig. 1e, Smarcd1 expression was dropped down to 13.1% and 10.1% while overexpressed up to 14.1- and 9.67-fold in U87 and U251 cells severally. After acquisition of 4 lentivirus-mediated stable transfection glioblastoma cell lines, namely knockdown control cell (kd-nc), knockdown Smarcd1 cell (kd-sm), overexpression control cell (over-nc), and overexpression Smarcd1 cell (over-sm), subsequent gene function analysis was conducted.

### Smarcd1 Inhibited the Proliferation of Glioblastoma Cells

**Fig. 2:**
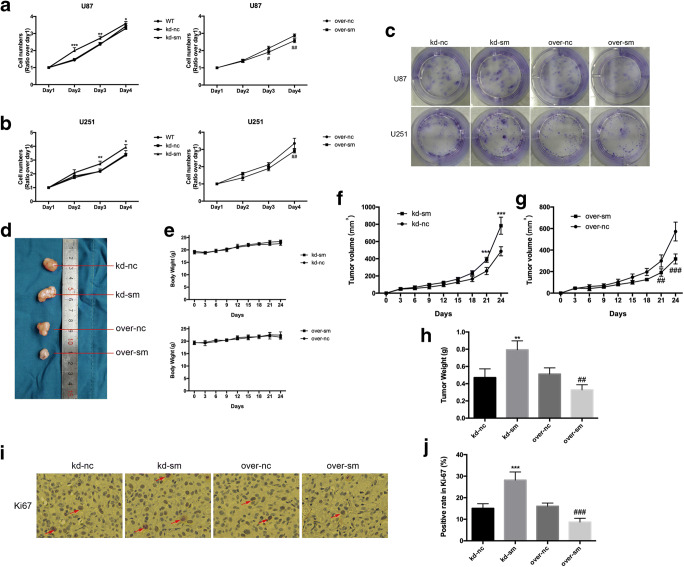
Smarcd1 inhibited glioblastoma cell growth both in vivo and in vitro. **a**, **b** Cell proliferation ability was detected by CCK-8 assay. Knockdown of Smarcd1 significantly promoted cell growth while overexpression of Smarcd1 showed the reverse function both in U87 (**a**) and U251 (**b**) cells. **c** Overexpression of Smarcd1 repressed the colony forming with decreasing colony number and size. **d**–**h** Tumor xenografts bearing U87 cells were taken to illustrate the role of Smarcd1 in vivo. Tumor volume (**f**) and weight (**h**) in the kd-sm group were significantly increased compared with the kd-nc group. Meanwhile, overexpression of Smarcd1 (**g** and **h**) suppressed the xenografts’ growth. **i**, **j** IHC assays showed that fewer Ki-67-positive cells in the over-sm group, which were quantified as significant difference (red arrows pointed out the Ki-67-positive cells). Data were represented as the means ± SEM of three independent experiments. *p < 0.05, **p < 0.01, ***p < 0.001 versus kd-nc group; #*p* < 0.05, ##*p* < 0.01, ###*p* < 0.001 versus over-nc group

To investigate the role of Smarcd1 in cell proliferation, we employed the CCK-8 assay to determine the tumor cell (U87 and U251) viability. The five groups of glioblastoma cell lines (wildtype, kd-nc, kd-sm, over-nc, over-sm) were seeded onto the 96-well plates and measured the OD450 values after cell adherence in 6 h (recorded as day 1) and then detected every 24 h for 3 consecutive times (until day 4). The results showed that U87 and U251 kd-sm groups were more proliferative than corresponding kd-nc groups (Fig. 2a); meanwhile, U87 and U251 cells which overexpressed Smarcd1 exhibited inverse results (Fig. 2b). Besides, we found that at the point of day 4 in U87 cells, the kd-sm group had slowed growth as compared to the kd-nc group, which may be due to the intensive attachment inhibition.

The long-term repressive role of Smarcd1 on U87 and U251 was determined by the colony-forming assay. A total of 1 × 10^3^ cells in each group suspended in the abovementioned culture medium were administrated to the 6-well plate and incubated for 12 days. As shown in Fig. 2c, the size and the number of colonies were much smaller in the over-sm groups than the control groups and kd-sm groups showed better colony-forming capacity.

Next, we intended to testify the prohibitive effects of Smarcd1 on glioblastoma growth in vivo; four groups of U87 lentivirus-transfected cells were harvested, and then, a total of 5 × 10^6^ cells were implanted subcutaneously in the flank of nude mice. As shown in Fig. 2d–h, the xenograft volume and weight were significantly inhibited in the over-sm group compared with the over-nc group. Inversely, glioma cells in the kd-sm group were prone to grow bigger than control, albeit no overt change in the total body weight of nude mice. The immunohistochemical staining of the xenograft sections revealed that Smarcd1 upregulation lowered the positive rate of Ki-67, which referred to enhanced cancer cell proliferation (Fig. 2 i and j). Taken together, Smarcd1 had a vital role in inhibiting glioblastoma growth.

### Smarcd1 Induced G1 Phase Arrest of the Cell Cycle in Glioblastoma Cells

**Fig. 3:**
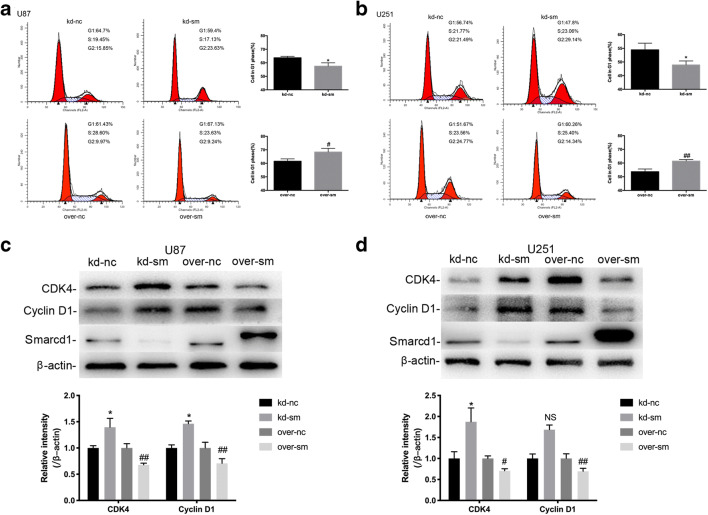
Smarcd1 induced G1 phase arrest. U87 (**a**) and U251 (**b**) glioma cells in the over-sm group exhibited significant higher proportion in G1 phase, which inhibited cell cycle progression. Glioma cells in the kd-sm group showed a decrease G1 phase. The expression of G1 phase arrest–related proteins, CDK4 and cyclin D1, were reduced after Smarcd1 overexpression in both U87 (**c**) and U251 (**d**) cells which was further confirmed by densitometric analysis of protein bands. The relative protein levels of control cells were adjusted to the value of 1. Data were represented as the means ± SEM of three independent experiments. *p < 0.05 versus kd-nc group; #*p* < 0.05, ##*p* < 0.01versus over-nc group

We then employed flow cytometry to evaluate the role of Smarcd1 in glioblastoma cell cycle for the reason that cell cycle arrest had a close correlation with suppressed cell propagation. As assumed, the percentage of over-sm cells in G1 phase was significantly increased than the over-nc groups in either U87 (Fig. 3a) or U251 (Fig. 3b) cells as long as G1 phase arrest always represented inhibition of cell growth. Subsequently, we examined the CDK4 and cyclin D1 protein levels, which directly regulated the G1 phase transition. The observations from the western blot bands illustrated that overexpression of Smarcd1 obviously decreased the levels of CDK4 and cyclin D1, while knockdown of Smarcd1 promoted CDK4 and cyclin D1 expression (Fig. 3c and d). However, quantitative analysis of cyclin D1 protein bands showed no significance in U251 cells (Fig. 3d), probably due to fewer repeated western blot detections and needed further measurements.

### Smarcd1 Infringed the Ability of Glioblastoma Migration and Invasion

**Fig. 4:**
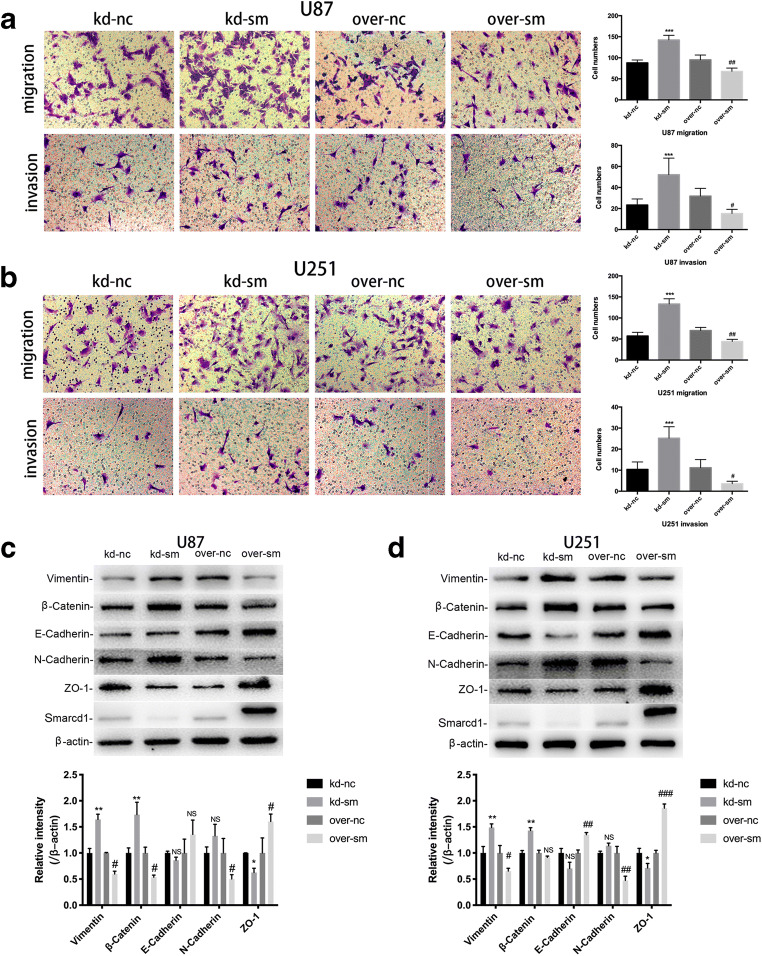
Smarcd1 suppressed the migration and invasion ability of glioblastoma cells. In transwell assays performed in U87 (**a**) and U251 (**b**) cells, the penetrated number of migrated and invasive cells was significantly reduced in the over-sm group compared with over-nc group. **c**, **d** The expression of pro-EMT proteins, including Vimentin, β-Catenin, and N-Cadherin, were suppressed in the over-sm group. The anti-EMT proteins, including E-Cadherin and ZO-1, were increased after overexpression of Smarcd1 in both U87 (**c**) and U251 (**d**) cell lines. Relative protein expression was counted with western blot band intensity by ImageJ software. The relative protein levels of control cells were adjusted to the value of 1. Data were represented as the means ± SEM of at least three independent experiments. *p < 0.05, *p < 0.01, ***p < 0.001 versus kd-nc group; #*p* < 0.05, ##*p* < 0.01, ###*p* < 0.001 versus over-nc group

Transwell assays were employed here to detect the motility of Smarcd1 upregulated or downregulated glioblastoma cells. After incubated in the upper chambers for 16 h, the four groups of penetrated cells were photographed and counted. We observed that overexpression of Smarcd1 impaired U87 and U251 cell migration and invasion than control, which were statistically significant. Meanwhile, the kd-sm groups showed increased migrated and invasive cells (Fig. 4a and b). Furthermore, we performed western blot to analyze some epithelial-mesenchymal transition (EMT) proteins, which were deemed as the crucial inducer of cell migration and invasion [20]. Vimentin, β-Catenin, and N-Cadherin are mesenchymal biomarkers which facilitate cell morphology change and motility. E-Cadherin and ZO-1 are epithelial biomarkers which reduce cell migration. Notably, Smarcd1 overexpression enhanced epithelial proteins, and after knockdown, the level of mesenchymal proteins upregulated in both U87 (Fig. 4c) and U251 (Fig. 4d) cells. All the protein bands were succumbed to densitometric analysis by ImageJ software with at least 3 different studies. Even though some quantitative results, such as E-Cadherin in U87 cells, showed no difference (Fig. 4c and d), we could also draw the conclusion that Smarcd1 inhibited the ability of invasion in glioma cells by combination of the transwell assays and the protein expression tendency.

### Smarcd1 Sensitized Glioblastoma to TMZ in P53-Dependent Pathway

Temozolomide (TMZ) is the first-line cytotoxic agent in the comprehensive treatment for glioblastoma. A number of patients suffer from TMZ chemoresistance partially due to hypomethylation of MGMT promoter [21]. Therefore, it is pivotal to discover gene targets to enhance chemotherapy efficiency for better GBM patients’ survival.

**Fig. 5:**
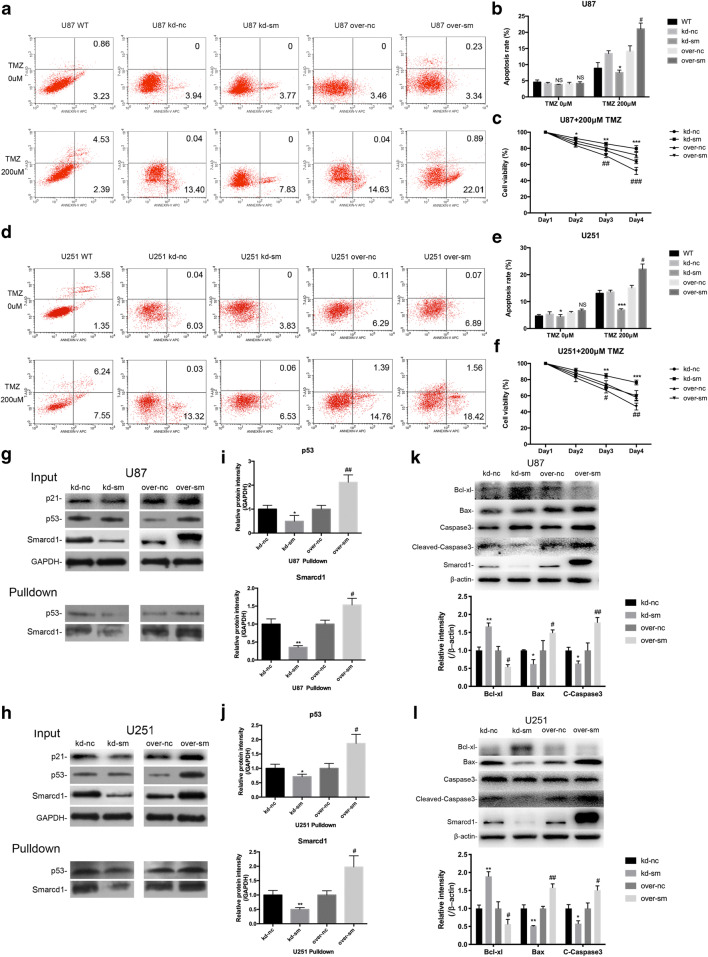
Smarcd1 overexpression promoted tumor apoptosis by interaction with P53 after TMZ treatment. **a**, **d** Flow cytometry was employed to detect cell apoptosis in U87 (**a**) and U251 (**d**) cells. **b**, **e** Quantification of apoptotic rates showed that Smarcd1 exerted little influence on cell apoptosis without TMZ treatment (referred as TMZ 0 μM). However, cells in the over-sm group showed higher apoptosis than those in the over-nc group, while those in the kd-sm groups exhibited fewer apoptosis rate after 200 μM administration. **c**, **f** The cell viability was analyzed by CCK-8 assay in U87 (**c**) and U251 (**f**) cell lines. Overexpression of Smarcd1 impaired cell viability after TMZ treatment in day 3 and day 4, while knockdown of Smarcd1 showed reverse results. **g**–**j** Smarcd1 could directly bind with P53 by Co-IP measurement. The quantification of pulldown proteins (**i**, **j**) showed that the binding P53 was positively related to the level of Smarcd1 in a significant manner. **k**, **l** The downstream proteins of the apoptotic cascade, Bcl-xl, Bax, and caspase3, were detected by western blot. The levels of Bax and Cleaved Caspase3 were increased while Bcl-xl was reduced in the over-sm group after TMZ treatment, which were further proven by densitometric analysis of the protein bands in U87 (**k**) and U251 (**l**) cells. The relative protein levels of control cells were adjusted to the value of 1. Data were represented as the means ± SEM of three independent experiments. *p < 0.05, **p < 0.01, ***p < 0.001 versus kd-nc group; #*p* < 0.05, ##*p* < 0.01, ###*p* < 0.001 versus over-nc group

Firstly, we performed flow cytometry assays to analyze the apoptotic rate in Smarcd1 knockdown and overexpression GBM cells in contrast to relative control cells. As shown in Fig. 5a, b, d, and e (referred as TMZ 0 μM), differential expression of Smarcd1 exhibited no obvious influence in GBM apoptosis except for the kd-sm group, which had a statistic difference than the kd-nc group of U251 cells (*P* < 0.05). Next, we investigated the role of Smarcd1 when treated with TMZ to determine whether Smarcd1 could influence GBM chemosensitivity. Different groups of lentivirus-mediated Smarcd1 expression cells were treated with 200 μM TMZ for certain time points and then detect tumor apoptosis and cell viability by flow cytometry and CCK-8 assays, respectively. Surprisingly, we observed that high expression of Smarcd1 showed more sensitivity to TMZ administration for 48 h with a significant increase in apoptotic rate than the control group in both U87 (Fig. 5b) and U251 (Fig. 5e) cell lines. GBM cells in the kd-sm groups had a lower apoptotic rate, which were resistant to TMZ (referred as TMZ 200 μM). Similarly, cancer cell viability in the over-sm group of the two cell lines was decreased while augmented in the kd-sm group after administration with TMZ for 48 h and 72 h (Fig. 5c and f). These results indicated that Smarcd1 overexpression rendered glioblastoma cells to TMZ toxicity, which could be a clinical predictor for chemotherapy efficiency.

As previously illustrated, Smarcd1 was authenticated to interact with P53 to recruit the SWI/SNF complex and then activate apoptosis cascade. Therefore, whether TMZ induced apoptosis in a P53-dependent manner had still to be uncovered. Immunoprecipitation assay was employed here to testify the direct combination between Smarcd1 and P53 in the condition of TMZ. As shown in Fig. 5g and h, knockdown of Smarcd1 led to a decreased expression of P53 and the downstream target P21 in the input U87 and U251 cell lysates, meanwhile the same tendency was found in overexpression of Smarcd1. With regard to IP pulldown products, the results confirmed that Smarcd1 could directly bind with P53 in a positive manner, which meant the more Smarcd1 expressed, the more P53 combined (Fig. 5g and h). Several repetitive assays showed the same results and quantified by ImageJ, which were statistically significant (Fig. 5i and j). Furthermore, we performed western blot studies to detect apoptosis-related proteins (Bcl-xl, Bax, and Caspase3) involved in the P53 pathway. The observations revealed that overexpression of Smarcd1 increased the expression of Bax and decreased Bcl-xl after the administration of TMZ, which promoted the transformation of Caspase3 to the Cleaved Caspase3, thus activating the apoptotic cascade. Quantification of the intensity of protein bands showed significant difference as mentioned above (Fig. 5k and l). Taken together, the above findings demonstrated that Smarcd1 could directly bind to P53 tumor suppressor protein and participate in the activation of P53 downstream genes, such as P21 and Bax. After knockdown of Smarcd1, P53 pathway was inactivated, which might stand reason to the decreased apoptosis and augmented chemoresistance of gliomas after TMZ treatment.

### The Crosstalk Between Smarcd1 and Notch1 Pathway Prompted Glioblastoma Malignancy

As previously mentioned, several subunits of the SWI/SNF complex, including BRG1, PB1, Hltf, and Snf2h, had been proven to function as cofactors for Notch1 transcriptional activity [17]. Smarcd1 is an indispensable subunit of the SWI/SNF family, and we accordingly hypothesized that Smarcd1 restrained glioblastoma cell proliferation and invasion potentially by regulation of Notch1 pathway.

**Fig. 6:**
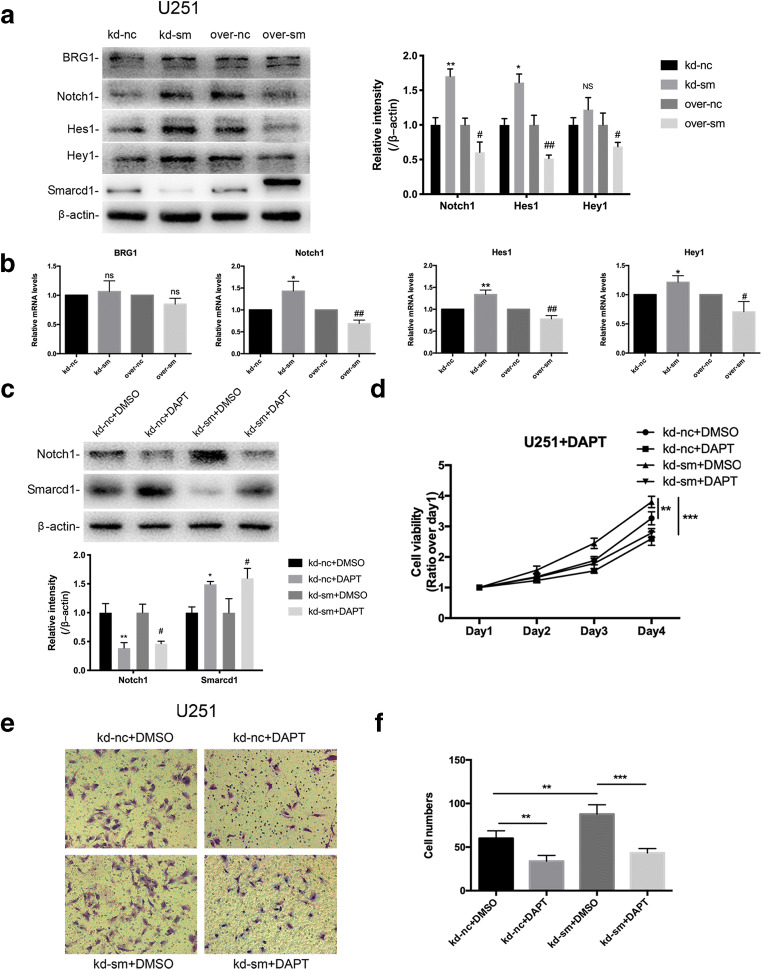
Smarcd1 inhibited the activation of Notch1 signaling. Knockdown of Smarcd1 augmented the protein (**a**) and mRNA (**b**) levels of Notch1, Hes1, and Hey1 in U251 cells. Differential expression of Smarcd1 had no influence on the level of BRG1 in protein expression (**a**) and transcription (**b**) levels. Relative protein expression was counted with western blot band intensity by ImageJ software. The relative protein levels of control cells were adjusted to the value of 1. Data were represented as the means ± SEM of three independent experiments. *p < 0.05, **p < 0.01, ***p < 0.001 versus kd-nc group; #*p* < 0.05, ##*p* < 0.01 versus over-nc group. Inhibition of Notch1 pathway by DAPT (**c**) restored the increased proliferation (**d**) and migration (**e**) abilities induced by knockdown of Smarcd1 in U251 cells. **f** Quantification of the migrated cells in transwell assays showed a significant decrease after DAPT treatment in both the kd-nc and kd-sm groups. *p < 0.05, **p < 0.01, ***p < 0.001 versus kd-nc + DMSO group; #*p* < 0.05 versus kd-sm + DMSO group

In consideration of the existing results above, U251 cell line exhibited a more representative glioma phenotype than U87, and hereafter, we only analyze U251 cells to clarify the potential mechanism which Smarcd1 regulated. Western blot and qPCR assays were performed to detect the expression of Notch1 and the downstream targets (Hes1 and Hey1). We observed that after knockdown of Smarcd1, the protein (Fig. 6a) and mRNA (Fig. 6b) levels of Notch1, Hes1, and Hey1 increased, while overexpression of Smarcd1 suppressed the activation of Notch1 pathway. Also, we found that BRG1, the catalytic subunit of SWI/SNF complex, was not overtly influenced when expression of Smarcd1 changed, which indicated that Smarcd1 induced the inactivation of Notch1 pathway which was independent of the cross-link to BRG1 (Fig. 6 a and b). Metaphysically, Smarcd1 could regulate Notch1 expression, but the concrete mechanisms underlying it needed further to be discovered.

DAPT (*N*-[*N*-(3,5-difluorophenacetyl)-L-alanyl]-S-phenylglycine t-butylester) is a γ-secretase specific inhibitor, which could suppress the formation of Notch intracellular domain (NICD), thus inactivating Notch1 pathway. DAPT was administrated to the kd-nc and kd-sm groups, and the results demonstrated that DAPT effectively inhibited the protein expression of NICD (Fig. 6c). As shown in Fig. 6d–f, knockdown of Smarcd1 increased the U251 cell viability and migration, which was consistent with Figs. 2a and 4b. However, treatment with Notch1 inhibitor DAPT restored the proliferation and migration abilities induced by low expression of Smarcd1, suggesting that downregulated Smarcd1 aggravated glioblastoma malignancy potentially via enabled Notch1 pathway.

**Fig. 7:**
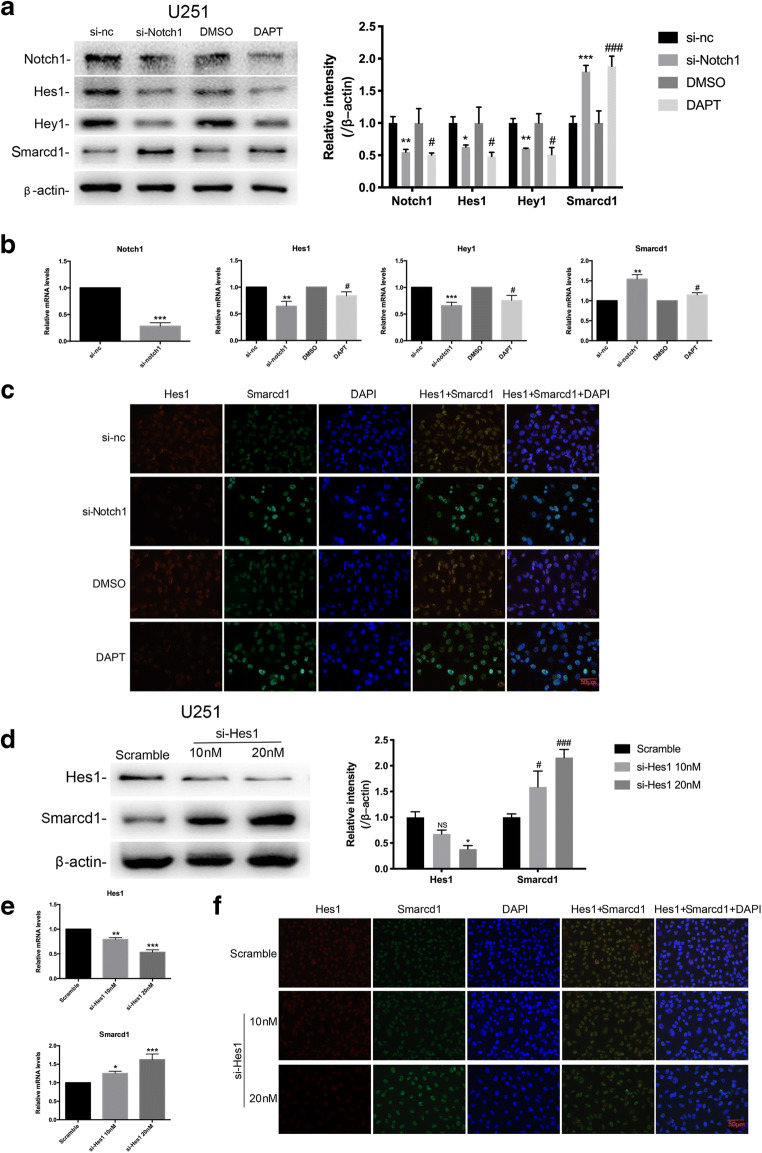
Inhibition of Notch1 increased Smarcd1 expression by suppression of Hes1 transcription. Inactivation of Notch1 pathway by Si-Notch1 and DAPT decreased the protein (**a**) and mRNA (**b**) levels of Hes1 and Hey1, meanwhile increasing the expression of Smarcd1 in U251 cells. Quantification of relative protein expression (**a**) was measured by ImageJ densitometric analysis, which showed significant difference as same as mRNA levels (**b**). The relative protein levels of control cells were adjusted to the value of 1. **p < 0.01, ***p < 0.001 versus si-nc group; #*p* < 0.05 versus DMSO group. **c** DAPT administration reduced the red fluorescence of Hes1 and brightened green fluorescence of Smarcd1. Knockdown of Hes1 mediated by siRNA increased the protein (**d**) and mRNA (**e**) levels of Smarcd1 measured by western blot band intensity and qRT-PCR. *p < 0.05, **p < 0.01, ***p < 0.001 versus scramble group. **f** The immunofluorescence density of Smarcd1 (green) was increased after si-Hes1 treatment, which was photographed by two investigators blind to the grouping. All data here were represented as the means ± SEM of three independent experiments

Nevertheless, we found an accordant increase of Smarcd1 protein level after DAPT treatment in Fig. 6c; therefore, we further testify whether Notch1 pathway regulated Smarcd1, thus forming a malignant loop. Notch1 siRNA and DAPT were utilized here to inhibit Notch1 activation, and then Hes1, Hey1, and Smarcd1 expression were detected. As expected, Si-Notch1 and DAPT administration in U251 cells significantly decreased the expression of Notch1 and targeted proteins while increased the Smarcd1 expression in both densitometric protein and mRNA levels (Fig. 7a and b). The immunofluorescence assay showed that inactivation of Notch1 reduced the Hes1 red fluorescence and heightened the density of Smarcd1 green fluorescence, where the merged figures show differential densities as we can distinguish compared to relative control groups (Fig. 7c). To investigate to potential underlying transcriptional factors, the promoter region (2000 bp upstream of first exon) of Smarcd1 was analyzed by the Jaspar database [22] (http://jaspar.genereg.net/). Surprisingly, we found several specific combining sites of Hes1, and then Hes1 siRNA (0 nM, 10 nM, and 20 nM) was administrated to U251 cells, respectively. As the dose of si-Hes1 increased, the expression of Hes1 declined while Smarcd1 expedited in a statistically significant manner (Fig. 7d and e), which indicated that Hes1 could serve as one of the transcriptional factors to regulate Smarcd1 expression. Immunofluorescence assay of Hes1 knockdown showed the same results (Fig. 7f). To sum up, the above findings manifested that Notch1 could inhibit the expression of smarcd1 by activating the transcription of Hes1, so there may be negative feedback crosstalk between Smarcd1 and Notch1.

## Discussion

Glioblastomas are lethal brain tumors with a 5-year survival rate reported less than 5.5% [23], which accounts for more than 50% of malignant gliomas [24]. Despite the novel GBM research frontier in immunotherapy and antiangiogenic treatments [25], efforts in finding biomarkers to predict the prognosis and to function as therapeutic targets are still crucial. In this work, main findings were concluded as follows: (1) Smarcd1 was less expressed in HGG and GBM cell lines than in normal brain tissues and NHA cells; (2) overexpression of Smarcd1 impaired tumor cell growth, induced cell cycle arrest and hinted cell migration; (3) Smarcd1 could directly bind with P53 and after TMZ treatment, upregulated Smarcd1promoted tumor cell apoptosis and suppressed cell viability in a P53-dependent manner; meanwhile, knockdown of Smarcd1 showed the reverse results; (4) the transcription and expression levels of Notch1, Hes1, and Hey1 were inhibited accompanying highly expressed Smarcd1. Inhibition of Notch1 could restore the enhanced proliferation and migration which were induced by knockdown of Smarcd1. And vice versa, Notch1 could repress the transcription of Smarcd1 via activation of Hes1, thus forming a Smarcd1-Notch1 negative feedback crosstalk.

Mammalian SWI/SNF complexes are combinatorial assemblies composed of at least 13 subunits encoded by 29 genes, which have been regarded as tumor suppressors in several human malignancies [26, 27]. More than 20% of human cancers reported are associated with mutations in SWI/SNF encoded genes [28]. The complexes use the energy of ATP hydrolysis to mobilize nucleosomes and remodulate genomic architecture, thus regulating transcription of target genes. Moreover, the pro- or anti-tumor effects of SWI/SNF complexes are complicated for the sake of discrepancies in cellular types, components and microenvironment. BRG1, which is the catalytic subunit of SWI/SNF, acts as a bona fide tumor suppressor in lung cancer [29], but shows an oncogene role in glioblastoma [30]. Besides, the stability of SWI/SNF subunits is affected by the interaction between each other, which makes the researches on SWI/SNF subunits even more intricate. Smarcd1 is a pivotal subunit of the SWI/SNF complex, and here, we found that differential expression of Smarcd1 exerted little effects on the possible oncogene BRG1, which indicated that Smarcd1 could function as a tumor suppressor independent of other subunits in the SWI/SNF family.

TMZ is an oral alkylating compound, which can penetrate the blood-brain barrier and can be applied as a first-line anti-glioma agent since approved in 2005 [31]. However, a number of patients are not sensitive to TMZ or even show resistance to TMZ with about only 45% efficiency as estimated [32]. The expression of MGMT mainly accounts for TMZ resistance and other factors including nucleotide base mismatch repair, base excision repair, P53 mutation and autophagy changes [33]. A previous study had demonstrated that Smarcd1 was involved in the regulation of tumor chemoresistance. In the cell model of lung cancer treated with cisplatin, miR-7 decoupled the interaction between Smarcd1 and P53 and then reduced P53-dependent downstream apoptosis cascades, thus promoting tumor chemoresistance [10]. Oh and his colleagues first reported that the activation of P53 pathway required the chromatin remodeling function of SWI/SNF complex, and only the subunit Smarcd1 directly interacted with P53 to form a bridge linkage between SWI/SNF and P53 in further research [6]. More concretely, the N-terminal residues of Smarcd1 could bind with the tetramerization domain of P53, and in LNCaP cell line, knockdown of Smarcd1 suppressed the transcriptional ability of P53, resulting in reduced P53-dependent tumor apoptosis [6]. The authors also found that overexpression of Smarcd1 was unable to promote P53 pathway activation. However, in our study, Smarcd1 overexpression alone did not induce tumor apoptosis, which was probably due to lack of interaction with P53. TMZ administration is affirmed to activate P53 pathway in glioblastoma. Hereafter with TMZ, Smarcd1 binds to adequate P53 and then facilitates the apoptotic cascade.

Notch signaling pathway is a multipotent regulator in cell fate decision, including lineage development, differentiation, cell cycle regulation and maintenance of cell stemness [34]. In glioblastoma, Notch1 pathway is involved in tumorigenesis and maintenance, and as a consequence, inhibition of Notch1 led to decreased cell viability, proliferation and a more differentiated morphology [35]. NICD (Notch intracellular domain) is a unique intermediate of Notch1 signaling in the transcription of downstream targets, of which NICD assembles chromatin remodulating proteins to regulate epigenetic modifications in the transcription start sites (TSS). In the present study, we also performed co-immunoprecipitation (Co-IP) assays to determine the association between Smarcd1 and NICD, but disappointedly, Smarcd1 could not capture NICD (data not shown). After literature reviewing, we realized that histone modifications (methylation or acetylation) are accomplished with Notch1 activation and suppression. The histone acetylases p300 can be recruited to NICD and then catalyze H3K27 into H3K27ac which facilitates chromatin loosening of the enhancer region and then stimulates transcription [36]. Histone demethylase Kdm5a catalyzes H3K4me3, which is considered to open chromatin structure, to the repressive form of H3K4 [37]. In addition, H3K27me3 also shows the relevance in close chromatin in NICD transcriptional process. Alajem and his colleagues reported that knockdown of Smarcd1 in embryonic stem cells redistributed H3K27me3 and H3K4me3 in the TSS of Klf4 gene [9]. Meanwhile, in HepG2 cells, the expression of Smarcd1 boosted the level of histone H3 acetylation and H3K4me3 proximal to Bmal1 promoter [8]. Taken together with aforementioned mechanisms, these may lend to the hypothesis that Smarcd1 could remodel the chromatin structure of Notch1 via specific modification of histones. ChIP-seq assays still need to delineate with detail the histone changes in the TSS regions of Notch1 pathway in further studies.

Furthermore, we demonstrated that inhibition of Notch1 decreased Smarcd1 expression by downstream targets Hes1 in the present study. Hes1 is a multipotent transcription repressive factor and plays a vital role in regulation of neural progenitor differentiation [38]. Silencing Hes1 expression in GSCs induced G1 phase arrest, inhibitory proliferation, and a more mature cell phenotype [39]. Hes1 protein contains bHLH domain and WRPW domain to participate in the combination of gene promoters. However, we only got an idea from the transcription factor database and performed the subsequent si-Hes1 experiments. Further investigation by luciferase reporter assays should be conducted to analyze the role of Hes1 in transcriptional regulation of Smarcd1 expression. The inner regulation mechanism between Smarcd1 and Notch1 is still an issue to be resolved.

Clinically, glioblastomas are highly heterogeneous with a subpopulation of glioma stem cells (GSCs), which result in tumor recurrence and TMZ chemoresistance [40]. Besides, the activation of Notch1 signaling, which is induced by low expression of Smarcd1, is capable to maintain cancer cell stemness. Therefore, it is reasonable that the crosstalk between Smarcd1 and Notch1 contributes to glioma chemoresistance potentially via the existence of GSCs, and strategies targeting pathways involving GSC to break up TMZ chemoresistance are the latest frontier of current GBM treatment.

To sum up, Smarcd1 was downregulated in glioblastoma tissues and cell lines. Overexpression of Smarcd1 inhibited tumor proliferation, migration and chemoresistance possibly via crosstalk with Notch1 pathway. Breaking up the malignant feedback loop between Smarcd1 and Notch1 may be a potential target in treating glioblastoma.

## Abbreviations

Smarcd1: SWI/SNF-related, matrix-associated, actin-dependent regulator of chromatin, subfamily d, member 1
CCK-8: Cell Counting Kit-8
PCR: Polymerase chain reaction
